# Involvement of the Modifier Gene of a Human Mendelian Disorder in a Negative Selection Process

**DOI:** 10.1371/journal.pone.0007676

**Published:** 2009-10-30

**Authors:** Isabelle Jéru, Hasmik Hayrapetyan, Philippe Duquesnoy, Emmanuelle Cochet, Jean-Louis Serre, Josué Feingold, Gilles Grateau, Tamara Sarkisian, Marc Jeanpierre, Serge Amselem

**Affiliations:** 1 INSERM, U933, Paris, France; 2 Université Pierre et Marie Curie-Paris6, UMR S_933, Paris, France; 3 Center of Medical Genetics and Primary Health Care, National Academy of Sciences, Yerevan, Armenia; 4 Equipe Structure-Fonction, EA 2493, Université de Versailles-Saint Quentin en Yvelines, Versailles, France; 5 INSERM U.567, Paris, France; Institut Pasteur, France

## Abstract

**Background:**

Identification of modifier genes and characterization of their effects represent major challenges in human genetics. *SAA1* is one of the few modifiers identified in humans: this gene influences the risk of renal amyloidosis (RA) in patients with familial Mediterranean fever (FMF), a Mendelian autoinflammatory disorder associated with mutations in *MEFV*. Indeed, the *SAA1* α homozygous genotype and the p.Met694Val homozygous genotype at the *MEFV* locus are two main risk factors for RA.

**Methodology/Principal Findings:**

Here, we investigated Armenian FMF patients and controls from two neighboring countries: Armenia, where RA is frequent (24%), and Karabakh, where RA is rare (2.5%). Sequencing of *MEFV* revealed similar frequencies of p.Met694Val homozygotes in the two groups of patients. However, a major deficit of *SAA1* α homozygotes was found among Karabakhian patients (4%) as compared to Armenian patients (24%) (p = 5.10^−5^). Most importantly, we observed deviations from Hardy-Weinberg equilibrium (HWE) in the two groups of patients, and unexpectedly, in opposite directions, whereas, in the two control populations, genotype distributions at this locus were similar and complied with (HWE).

**Conclusions/Significance:**

The excess of *SAA1*α homozygotes among Armenian patients could be explained by the recruitment of patients with severe phenotypes. In contrast, a population-based study revealed that the deficit of α/α among Karabakhian patients would result from a negative selection against carriers of this genotype. This study, which provides new insights into the role of *SAA1* in the pathophysiology of FMF, represents the first example of deviations from HWE and selection involving the modifier gene of a Mendelian disorder.

## Introduction

Interest in modifier genes keeps growing because of their ability to modulate the expression of monogenic or multigenic traits and diseases [1], [2]. *SAA1*, which encodes the serum amyloid A protein, is one of the few modifier genes identified in humans [3]. Three *SAA1* allelic variants have been defined on the basis of two single nucleotide polymorphisms located in exon 3 (c.209C>T–p.Ala52Val and c.224T>C–p.Val57Ala): α (c.209T, c.224C), β (c.209C, c.224T) and γ (c.209C, c.224C). We previously demonstrated that the *SAA1* α/α genotype is associated with an increased risk to develop renal amyloidosis (RA) in Armenian patients suffering from familial Mediterranean fever (FMF [MIM 249100]) [3], a result that was secondarily confirmed in other populations of FMF patients [4]–[7]. FMF, which belongs to the expanding family of autoinflammatory disorders, is an autosomal recessive disorder associated with mutations in the *MEFV* gene [8], [9]. The effect of the *SAA1* α/α genotype on the severity of the disease phenotype, therefore, reveals an epistatic interaction between *SAA1* and *MEFV*. FMF is characterized by recurrent episodes of fever and systemic inflammation, usually revealed by sterile peritonitis, arthritis and/or pleurisy. During attacks, patients have elevated levels of acute phase reactants, especially of SAA, that leads in some patients to RA as a result of renal deposition of protein amyloid A. This reactive systemic amyloidosis, called AA-amyloidosis, which may develop over years and progress to terminal renal failure, is the major complication of FMF [10].

FMF primarily affects people originating from the Mediterranean basin, especially Armenian, Sephardic Jewish, Turkish and Arab populations. The prevalence of FMF in Armenia and in Karabakh, a region close to Armenia inhabited by Armenian people, is particularly high ranging from 0.5 to 1.5% [11]. Several studies, performed before the introduction of colchicine treatment, reported different prevalences of RA in Armenian FMF patients living in different countries: 24% in Armenia [12], 7.5% in Lebanon [13] and 0% in California [14]. However, to date there has been no explanation for this observation. Very recently, it has been noted that RA is rare among untreated Karabakhian patients (2.5%, Sarkisian, unpublished observations), as compared to untreated patients living in Armenia (24%, [12], Sarkisian, unpublished data), therefore suggesting the presence of a protective factor in Karabakh.

## Results and Discussion

### Difference in *SAA1* genotype distributions between Armenian and Karabakhian patients

The difference in the prevalence of FMF-associated renal amyloidosis in patients from Armenia and from Karabakh prompted us to compare, in these two populations, the genetic factors known to influence the risk to develop RA: the p.Met694Val homozygous genotype at the *MEFV* locus (disease-causing gene) and the α homozygous genotype at the *SAA1* locus (modifier gene), each of these two factors being associated with a sevenfold increased risk for RA [3]. Sequencing of *MEFV* did not reveal any significant difference in the frequency of p.Met694Val homozygotes between Armenian and Karabakhian patients (Table 1). However, sequencing of *SAA1* revealed that the frequency of FMF patients with the α/α genotype was much higher in Armenia (f = 0.24) than in Karabakh (f = 0.04) (p = 5.10^−5^, 1d.f.) (Table 2). At first glance, these data suggest that the low frequency of the α/α genotype among patients from Karabakh could account for the low prevalence of amyloidosis in that region. These observations led us to investigate in more details the role of the *SAA1* modifier in patients from Armenia and from Karabakh, as well as in matched population controls.

**Table 1:** Comparison of the *MEFV* p.Met694Val homozygous genotype frequencies in Armenian and Karabakhian FMF patients.

*MEFV* genotype	Armenian patients No. (%)	Karabakhian patients No. (%)	p-value
p.Met694Val/p.Met694Val	23 (0.23)	30 (0.3)	
other	77 (0.77)	69 (0.7)	
			0.24

**Table 2:** Comparison of the *SAA1* α homozygous genotype frequencies in Armenian and Karabakhian FMF patients.

*SAA1* genotype	Armenian patients No. (%)	Karabakhian patients No. (%)	p-value
α/α	24 (0.24)	4 (0.04)	
other	76 (0.76)	95 (0.96)	
			5.10^−5^

### Opposite deviations from HWE of *SAA1* genotype distributions among Armenian and Karabakhian patients

**Figure 1:**
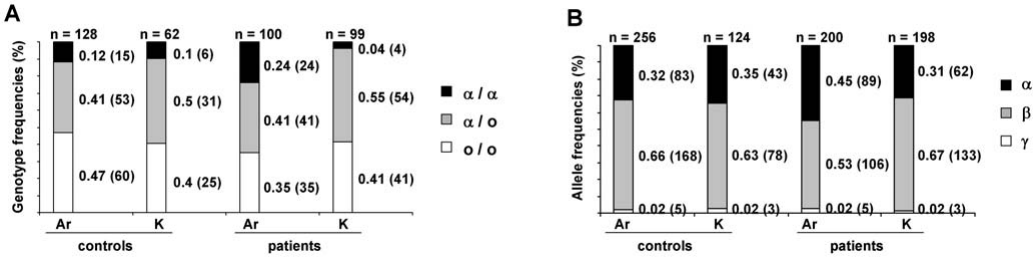
*SAA1* genotype and allele distributions in Armenian and Karabakhian FMF patients and controls. A. Genotype frequencies. B. Allele frequencies. Ar = Armenia, K = Karabakh. o stands for “other allele” that includes β and γ alleles; since the frequency of the γ allele is very low in Armenian individuals, β and γ alleles were pooled for statistical analyses. Numbers in brackets correspond to the numbers of individuals in each category.

**Figure 2:**
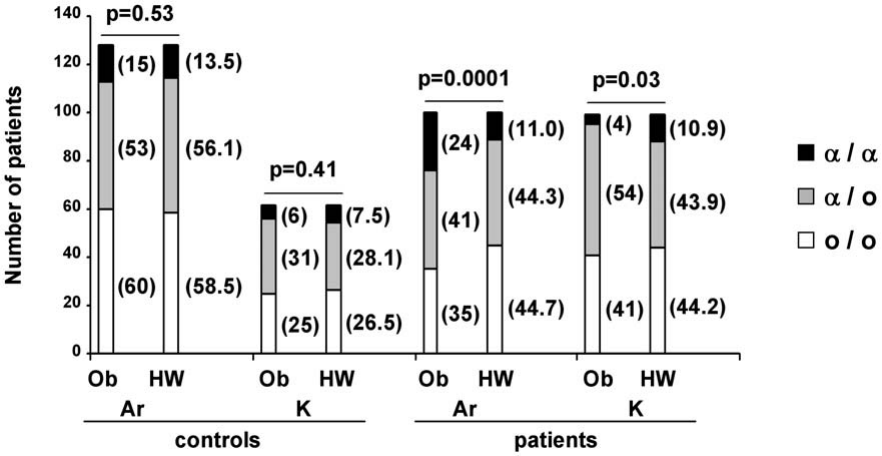
Comparison of *SAA1* genotype distributions to theoretical proportions expected from HWE. Ob = observed, HW = expected under HWE, Ar = Armenia, K = Karabakh. In the two groups of patients, the genotype distributions expected under HWE were calculated using the allele frequencies of the control group; in that case, we used a chi-square test with 2 degrees of freedom.

We first investigated the genotype and allele distributions at the *SAA1* locus in Armenian and Karabakhian control groups (Fig. 1A and 1B). We observed that the most frequent *SAA1* alleles correspond to α and β variants (frequency in Armenia of 0.32 and 0.66, respectively; frequency in Karabakh of 0.35 and 0.63, respectively), whereas the γ allele is very rare (frequency in Armenia and Karabakh of 0.02). Since α is the *SAA1* allele involved in the risk to develop RA in Armenian FMF patients, β and γ alleles were merged into a single allele category termed “o” (standing for other alleles) in subsequent statistical analyses. Then, we compared the distribution of *SAA1* genotypes in controls from Karabakh and Armenia (Fig. 1A) by means of a chi-square test of homogeneity. No difference was observed (p = 0.53, 2d.f., Fig. 2), a result allowing to consider the Karabakhian and Armenian controls as a single homogeneous control group. In addition, as shown by chi-square tests, these distributions at the *SAA1* locus fit with Hardy-Weinberg equilibrium (HWE) in the two control groups (Karabakh: p = 0.53, Armenia: p = 0.41, 1 d.f.) (Fig. 2).

**Figure 3:**
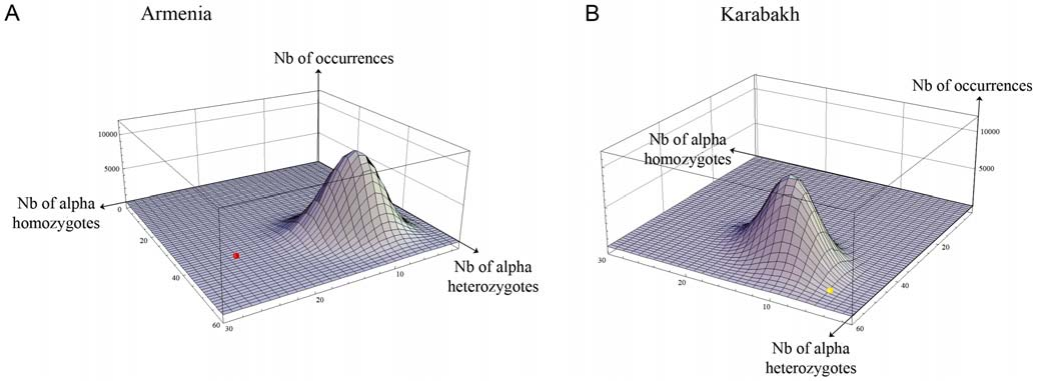
Comparison of *SAA1* genotype distributions to sampling fluctuations in populations under the assumption of HWE. A. Comparison in Armenian patients. A Monte Carlo algorithm was used to represent the data from 10^6^ simulations of *SAA1* genotype distributions in samples of 100 individuals assuming that the α allele frequency is 0.32 (i.e. frequency in the Armenian control group). The 3D representation shows the number of occurrences for each genotype distribution (z-axis) according to the number of α homozygotes and α heterozygotes (x- and y-axis, respectively) in a Cartesian coordinate system. The position of the genotype distribution observed in Armenian patients is plotted as a red dot (position 24;41). B. Comparison in Karabakhian patients. Same as in A with the representation of the data from 10^6^ simulations in samples of 99 individuals, assuming that the α allele frequency is 0.35 (i.e. frequency in the Karabakhian control group). The position of genotype distribution observed in Karabakhian patients is plotted as a yellow dot (position 4;54).

However, a similar analysis performed in the population of FMF patients living in Armenia revealed that the distribution of *SAA1* genotypes does not comply with HWE (p = 1.10^−4^, 2d.f., Fig. 2), and that the frequency of the α/α genotype is much higher in patients (f = 0.24) than in controls (f = 0.11) (Fig. 1A) (p = 4.10^−3^, 1d.f.). The study of the distribution of *SAA1* genotypes among Karabakhian patients also showed a departure from HWE (p-value for the chi-square test p = 0.03, 2d.f., Fig. 2), but, unexpectedly, with a lower frequency of α homozygotes in patients (f = 0.04) than in controls (f = 0.1) (Fig. 1A, p = 0.04, 1d.f.). Consequently, the frequency of α homozygotes differs dramatically between Karabakhian (f = 0.04) and Armenian patients (f = 0.24) (Fig. 1A, p = 5.10^−5^, 1d.f.). To further confirm these data, we compared the distributions of *SAA1* genotypes observed in Armenian and Karabakhian patients to the fluctuations expected in samples under the assumption of HWE, using a Monte Carlo algorithm. Indeed, for large samples (n>30), Monte Carlo simulations, which are easy statistical tools to represent variations due to stochastic fluctuations, are more appropriate than chi-square tests to determine whether a given population follows the Hardy-Weinberg law [15], [16]. Based on the frequency of the different *SAA1* alleles calculated in the Armenian and Karabakhian groups of patients, we performed 10^6^ random simulations of the *SAA1* genotype distributions expected under HWE in samples of 100 and 99 individuals, respectively. We represented the probability of each genotype distribution on a three-dimensional (3D)-graph displaying the number of α homozygotes (x-axis) as a function of the number of α heterozygotes (α/o) (y-axis) and the number of occurrences for each genotype distribution (z-axis) (Fig. 3). Since we used a high number of simulations, sampling variations are represented as a 3D-Gaussian curve. As shown in Fig. 3A and 3B, the *SAA1* genotype distributions observed in the Karabakhian and Armenian groups of patients correspond to very rare events (dots located at far extremities of the 3D curves). This second independent statistical approach therefore confirmed major and opposite departures from HWE in the group of FMF Armenian patients (p<5.10^−5^) that showed a large excess of α homozygotes, and in the group of Karabakhian patients (p<5.10^−4^) that displayed a major lack of α homozygotes.

At this point, it is important to underline the fact that correct interpretation of HWE deviations depends on the reliability of genotyping. In this regard, and as detailed in the Materials and Methods section, we followed a number of experimental safeguards to ensure its accuracy. In addition, the fact that HWE deviations were absent in control groups and found in opposite directions in the Armenian and Karabakhian populations of patients also strongly argues against genotyping errors, all the more since all samples (from Armenian and Karabakhian individuals) were processed at the same time and following identical experimental procedures.

### Negative selection against *SAA1* α/α carriers

How the deviation from HWE observed in Armenian patients could be explained? Family history of each patient revealed no consanguinity that could account for such a deviation. Genetic drift can also be excluded since the distributions of *SAA1* genotypes complied with HWE in the Armenian control group. Therefore, the most likely explanation for this increase in *SAA1* α homozygotes among patients is the recruitment of severe forms of the disease, thereby underlining the prognostic value of the *SAA1* α/α genotype [3]–[7]. As for the deviation from HWE observed in the Karabakhian population of patients, it was, in that case, associated with a deficit of *SAA1* α/α. Consanguinity was also ruled out on family history; in addition, consanguinity would have led to an increase of homozygotes, a situation opposite to the one observed. A selection bias can be excluded because it would have favored the recruitment of patients with severe phenotypes and, as discussed above, it would have increased the frequency of α homozygotes. Genetic drift can also lead to HWE departures in populations of limited size due to random fluctuations of allelic frequencies from one generation to the next one [17]. However, the fact that the HWE disequilibrium observed in Karabakh was found only in the subset of FMF patients and not in controls discards this hypothesis. Mixed populations is another common cause of deviation from HWE since population substructure leads to a reduction of heterozygotes, a phenomenon called the Wahlund effect [18]; however, in the present study, the proportion of α heterozygotes is not reduced but even higher than expected, thereby strongly arguing against population substructure. The only reasonable interpretation for the observed departure from HWE is therefore selection against FMF patients carrying the α homozygous genotype. This conclusion is also supported by the fact that this particular *SAA1* genotype has been shown to be a major severity factor of the disease in all populations investigated so far [3]–[7]. The negative selection would occur very early, possibly leading to abortion, since no case of early death has been observed among Karabakhian patients with FMF. Given the prevalence of FMF in Karabakh (∼0.01) and the frequency of the alpha/alpha genotype in this area (∼0.1), the expected percentage of Karabakhian alpha homozygous FMF patients is very low (at most 1.10^−3^); a negative selection could, therefore, easily pass unnoticed. The study of the Karabakhian history reveals that Karabakhian and Armenian populations have evolved relatively independently since several centuries [19]; consequently, and as an example, it has been demonstrated that the spectrum of *MEFV* mutations (nature and frequency) differs in the two populations, although the frequency of the particular p.Met694Val homozygous genotype, the one that increases the risk of renal amyloidosis, is similar among Karabakhian and Armenian patients [20], [21]. In this context, we wondered whether a particular *SAA1* allelic variant could be present in Karabakh. Sequencing of the entire *SAA1* gene revealed 53 polymorphisms (including the 2 polymorphisms defining the α, β and γ alleles), but none of them was found more frequently in Karabakhian than in Armenian individuals (data not shown). It is therefore likely that the lack of *SAA1* α homozygotes in Karabakh brings into play other as-yet-unknown genes and/or environmental factors.

Identification of modifier genes and characterization of their associated effects represent one of the main and most difficult current challenges in human genetics. In the present study, we identified a new characteristic of the *SAA1* modifier. We observed a major difference in the distribution of *SAA1* genotypes in two groups of patients from the same origin but living in neighboring countries, though no difference was observed in matched control groups. Furthermore, we observed significant deviations from HWE in the two groups of patients, but strikingly in opposite directions. Our data strongly suggest that *SAA1* not only influences the severity of FMF, but is also involved in a negative selection process against Karabakhian FMF patients carrying the *SAA1* α/α genotype. Very few studies have reported the involvement of disease-causing or susceptibility genes in negative selection processes in humans [22]–[25]. To the best of our knowledge, this is the first study showing deviations from HWE and selection involving a modifier gene involved in a Mendelian disorder.

## Materials and Methods

### Subjects

The study was approved by the Institutional Review Board of the Ministry of Health (Republic of Armenia). We investigated consecutive unrelated patients originating from Armenia (n = 100) and from Karabakh (n = 99) who came to hospital and in whom the diagnosis of FMF was made according to established clinical criteria [26]. Clinical features were recorded through a standardized form and informed written consent was given by all individuals. 128 healthy individuals from Armenia and 62 from Karabakh were investigated as controls. All individuals are of Armenian descent. Each study group consists of individuals that are not related to the first, second or third degree.

### Screening of the *MEFV* and *SAA1* genes

Mutations in *MEFV* were screened as described previously [3]. The *SAA1* gene was amplified by PCR (Expand Long Template PCR System, Roche) from genomic DNA using forward (5′-tgacctgcagggactttccccagg-3′) and reverse (5′-aactcatcatgtccttgcctcagg-3′) primers. PCR products, run on agarose gels together with a negative control to detect any possible contamination, were then purified using the Wizard PCR Clean-Up System (Promega), sequenced with both forward and reverse primers using the ABI PRISM Big Dye Terminator V3.1 ready reaction cycle sequencing kit, and processed on a Genetic Analyzer ABI 3100 (Applied Biosystems). Electrophoregrams, analyzed by a dedicated software, were also read independently by two persons. Samples from Armenian and from Karabakhian individuals were processed at the same time using identical experimental conditions.

### Statistical analyses

Pearson's chi-square tests were used to compare the frequencies of p.Met694Val homozygotes and *SAA1* α/α homozygotes in the different groups. The compliance with HWE of *SAA1* genotype distributions was also tested using chi-square tests. In the 2 groups of patients, genotype distributions expected under HWE were calculated using the allele frequencies of the control group including Armenian and Karabakhian controls; compliance with HWE was assessed using chi-square tests with 2 degrees of freedom. We used a Monte Carlo algorithm to represent in a 3D model the expected variations of *SAA1* genotype distributions due to stochastic sampling fluctuations in populations under the assumption of HWE: iterative (n = 10^6^) sample simulations of sizes matching those of the two groups of patients (n = 100 for Armenia and n = 99 for Karabakh) were performed using the *SAA1* allele frequencies estimated in the corresponding control groups (f(α) = 0.32 and 0.35 in Armenia and Karabakh, respectively). The number of occurrences for each genotype distribution is represented in a Cartesian coordinate system, the juxtaposition of all simulations leading to a 3D Gaussian curve.
